# Transparency of AI in Healthcare as a Multilayered System of Accountabilities: Between Legal Requirements and Technical Limitations

**DOI:** 10.3389/frai.2022.879603

**Published:** 2022-05-30

**Authors:** Anastasiya Kiseleva, Dimitris Kotzinos, Paul De Hert

**Affiliations:** ^1^LSTS Research Group (Law, Science, Technology and Society), Faculty of Law, Vrije Universiteit Brussels, Brussels, Belgium; ^2^ETIS Research Lab, Faculity of Computer Science, CY Cergy Paris University, Cergy-Pontoise, France

**Keywords:** transparency, interpretability, explainability, artificial intelligence (AI), accountability, healthcare, informed medical consent, medical devices

## Abstract

The lack of transparency is one of the artificial intelligence (AI)'s fundamental challenges, but the concept of transparency might be even more opaque than AI itself. Researchers in different fields who attempt to provide the solutions to improve AI's transparency articulate different but neighboring concepts that include, besides transparency, explainability and interpretability. Yet, there is no common taxonomy neither within one field (such as data science) nor between different fields (law and data science). In certain areas like healthcare, the requirements of transparency are crucial since the decisions directly affect people's lives. In this paper, we suggest an interdisciplinary vision on how to tackle the issue of AI's transparency in healthcare, and we propose a single point of reference for both legal scholars and data scientists on transparency and related concepts. Based on the analysis of the European Union (EU) legislation and literature in computer science, we submit that transparency shall be considered the “way of thinking” and umbrella concept characterizing the process of AI's development and use. Transparency shall be achieved through a set of measures such as interpretability and explainability, communication, auditability, traceability, information provision, record-keeping, data governance and management, and documentation. This approach to deal with transparency is of general nature, but transparency measures shall be always contextualized. By analyzing transparency in the healthcare context, we submit that it shall be viewed as a system of accountabilities of involved subjects (AI developers, healthcare professionals, and patients) distributed at different layers (insider, internal, and external layers, respectively). The transparency-related accountabilities shall be built-in into the existing accountability picture which justifies the need to investigate the relevant legal frameworks. These frameworks correspond to different layers of the transparency system. The requirement of informed medical consent correlates to the external layer of transparency and the Medical Devices Framework is relevant to the insider and internal layers. We investigate the said frameworks to inform AI developers on what is already expected from them with regards to transparency. We also discover the gaps in the existing legislative frameworks concerning AI's transparency in healthcare and suggest the solutions to fill them in.

## Introduction

According to the European Commission, one of the ways that artificial intelligence (AI) will change our lives is by improving the healthcare (European Commission, 2020). Powered by the increasing availability of healthcare data and the rapid progress of analytical techniques (Kiseleva, 2020a), AI has enormous potential in improving the diagnosis and the clinical care, in enhancing the health research and drug development, increasing the efficacy of resource allocation, and improving the healthcare management (WHO Guidance, 2021, p. V).

The potential of AI in healthcare stimulates the technological giants investing in the field^1^ and gives rise to new medical and tech start-ups. To redeem their investments and expenses, the companies need to have the ability to place their technologies on the market and commercially use them. For that, the AI-based applications have to go through the relevant authorization procedures by the controlling bodies^2^. Despite strong questioning of policymakers and scholars if the existing legal frameworks are prepared for AI, the amount of AI medical applications already approved under these frameworks is expanding. For the period between 2015 and 2020, Urs J Muehlematter et al. identified that 222 AI-based medical devices^3^ were approved in the USA and 240 devices in Europe (Muehlematter et al., 2021). The range of medical fields covering the approved applications is rather broad and includes, for example, clinical toxicology, gastroenterology, molecular genetics, ophthalmology, microbiology, hematology, anesthesiology, neurology, and cardiovascular (Muehlematter et al., 2021). However, radiology holds the absolute championship in the amount of approved AI-based medical devices. For example, as of December 2021, the number of AI applications in radiology and related fields in the USA since 2008 is 151 (AI Central, 2021).

At the same time, policymakers around the world are concerned about the legal and ethical challenges posed by AI. One of the main issues is the lack of AI's transparency. Since the very first promising results of AI in healthcare, the “black-box” problem [opacity of how AI comes to decisions (Linardatos et al., 2021)] became a cornerstone of its successful application in clinical practice^4^. While the lack of algorithmic transparency is an issue in itself, in healthcare, it is crucial because people's lives and health are at stake. In many cases but especially if something goes wrong, we have to know why and how to prevent it in the future. For that, we need to trace how the algorithmic input turned into the specific output and what were factors that contributed to it. Yet, in the case of AI, this is not always possible.

In April 2021, the European Commission issued the first legislative proposal to regulate AI at the EU level—the Proposal for a Regulation of the European Parliament and of the Council laying down the harmonized rules on AI [EC Proposal for the AI Act (European Commission, 2021)]. The proposed AI Act intends to mainly cover high-risk AI systems – the ones that pose significant risks to the health and safety or the fundamental rights of persons (EC proposal for the AI Act, p. 3). AI applications that are used in healthcare for medical purposes are classified as high risk and will have to comply with two frameworks – the future AI Act and the Medical Devices Framework (MDF). The MDF consists of the Medical Devices Regulation [MDR (Regulation, 2017a)] and the *In-Vitro* Medical Devices Regulation [IVDR (Regulation, 2017b)] and is already applicable for placing AI medical devices on the market^5^.

For AI-based medical devices, transparency will be one of the core requirements as soon as the AI Act is adopted. Under the requirement, an AI system is deemed sufficiently transparent if it enables its users to interpret the AI's system output and apply it appropriately [EC Proposal for the AI Act, art 13(1)]. Yet, what exactly interpretation means and what are the relevant measures that are acceptable by the legislator are still open questions. Considering the variety of views on transparency, interpretability, and explainability among scholars in both legal and technical domains, this legislative ambiguity might cause difficulties to AI developers in compliance.

Before the relevant legislation is adopted, the research on how transparency of AI is perceived in both legal and technical domains can contribute to the development of the common interdisciplinary taxonomy. In this paper, we develop a *single point of reference* for both legal scholars and data scientists on transparency and related concepts. For that, in Section AI's Transparency: Between Law and Computer Science we first analyze how transparency stands in the current EU legislation^6^ and what kind of behavior is usually associated with it. We further explore the views of data scientists on transparency, interpretability, and explainability of AI and the correlation between all these concepts. For that, we analyze the literature with the focus on taxonomy and terminology discussions in the field^7^. Based on the findings of Section AI's Transparency: Between Law and Computer Science, we suggest that AI's transparency shall be viewed as the system of measures and as a continuous process accompanying the whole life cycle of an AI application.

The approach to view the transparency as the system of measures rather than one single obligation shall be considered the basis for any development and use of AI. However, a transparency system can start functioning properly only when it is placed in the context. The context defines involved subjects, their expectations with regards to transparency, as well as the legal frameworks already regulating relations between them. All these factors are essential for shaping the transparency-related rights and obligations. In Section Functions and Types of AI's Transparency in Healthcare, we place the analysis of transparency in healthcare context. We define what functions the transparency generally performs in healthcare, and we submit the ones applicable for AI: accountability; ensuring safety and quality; enabling making informed choices. We also explore how transparency in healthcare is classified based on addressees and addressors of transparency. We suggest that the multilayered system of transparency shall be built through accountability by defining who, how, and when shall do what to whom with regards to transparency. This accountability picture relevant to transparency shall be part of the general accountability system established in the specific domain. Risk management is part of accountability, and we explore how this factor shall be considered in healthcare for dealing with the algorithmic opacity.

Our multilayered transparency system concerns the main groups of the subjects involved in the life cycle of AI medical applications – starting from its developers to healthcare professionals and finally patients. Based on that, we classify transparency in two types (depending on its addressees): external (toward patients as the subjects who are not the part of the healthcare system but its clients) and internal (toward healthcare providers – subjects who are part of the system). As part of the internal transparency, we also distinguish the insider transparency – the one from AI developers toward themselves. Based on these types of AI's transparency, we will analyze the applicable legal frameworks [informed medical consent requirement (in Section External Transparency of AI in Healthcare: Toward Patients) and the MDF (in Sections Internal Transparency of AI in Healthcare: Toward Healthcare Providers and Insider Transparency of AI in Healthcare: Toward AI Developers)] to see what is expected from the transparency actors, if it is sufficient in AI context and if not, what else shall be added. In the final section (Section Summary and Discussions), we summarize the findings and suggestions.

This paper provides several contributions. First, it makes the interdisciplinary analysis of AI's transparency and suggests its correlation with the neighboring concepts such as explainability, interpretability, information provision, traceability, auditability, records keeping, documentation, data governance, and management. The suggested correlation establishes that transparency shall be viewed as the highest concept achieved through the said neighboring concepts. Second, the vision of transparency as a multilayered system of accountabilities makes it the common goal for all the stakeholders involved, not only the burden of the AI providers. At the same time, broadening the scope of the transparency-related measures gives the relevant subjects (including AI developers) more tools to demonstrate their legal compliance and to achieve their expectations from transparency. Finally, we analyze the legal frameworks applicable for the use of AI in healthcare to inform the AI developers on what is already expected from them with regards to transparency and what else might be needed to build the context-specific AI's transparency system through accountability tools. In this context, we discover the gaps in the existing legislative frameworks and suggest the solutions to fill them in.

## AI's Transparency: Between Law and Computer Science

### Views on AI's Transparency: Law and Policy

#### Transparency in the Primary and Secondary EU Legislation

Although the term transparency is not new for the EU legal system, it has gained special attention over the recent EU legislative activities related to the regulation of new technologies. In the era of the information society (Webster, 2006), access to information is the main asset and at the same time is the empowering mechanism. Often, the information is in the hands of developers of the technologies that collect, analyze, and produce information which creates an unequal distribution of this asset. The law has to address these issues. Several legislative activities in recent years deal with informational asymmetries. These activities include adopting the General Data Protection Regulation [GDPR (Regulation, 2016)] and discussions around a regulatory framework for AI. Because of that, transparency is becoming more popular and more distinguished in a legal sense.

In this section, we aim to analyze how transparency stands in the EU legislation. To reach the aim, rather than developing the overarching definition of transparency, we focus on discovering and summarizing what kind of actions are usually associated with the term “transparency” in the current EU legal acts.

We analyze the primary [CFR (Charter of Fundamental Rights of the European Union, 2012)] and secondary EU legislations. Secondary legislation is chosen in the EUR-Lex database (Eur-Lex, 2021) based on the inclusion of the word “transparency” either in the title of the law or in its text. The selection of the sources is not all encompassing. Our analysis only aims to provide a summarized picture on transparency rather than a full one.

The EU CFR does not explicitly use the term “transparency.” However, it contains terms and concepts close to transparency: the requirement of informed medical consent (CFR, art. 3); the right to receive information and ideas (CFR, art. 11); the workers' right to information and consultation (CFR, art. 27); the right of every person to have access to his or her file as part of the right to good administration (CFR, art. 41); and the right of access to the EU Parliament's, Council's, and Commission's documents (CFR, art. 42). These rights vary in scopes and forms, but all of them refer to the availability of information (or of documents as its specific form). Although transparency is wider than access to information and might contain other elements as explained further, access to information is its crucial element^8^. While the primary legislation establishes the fundamental legal concepts, protection at this level of the main transparency's element - *the right to information*- is important for its legal recognition.

**Figure 1:**
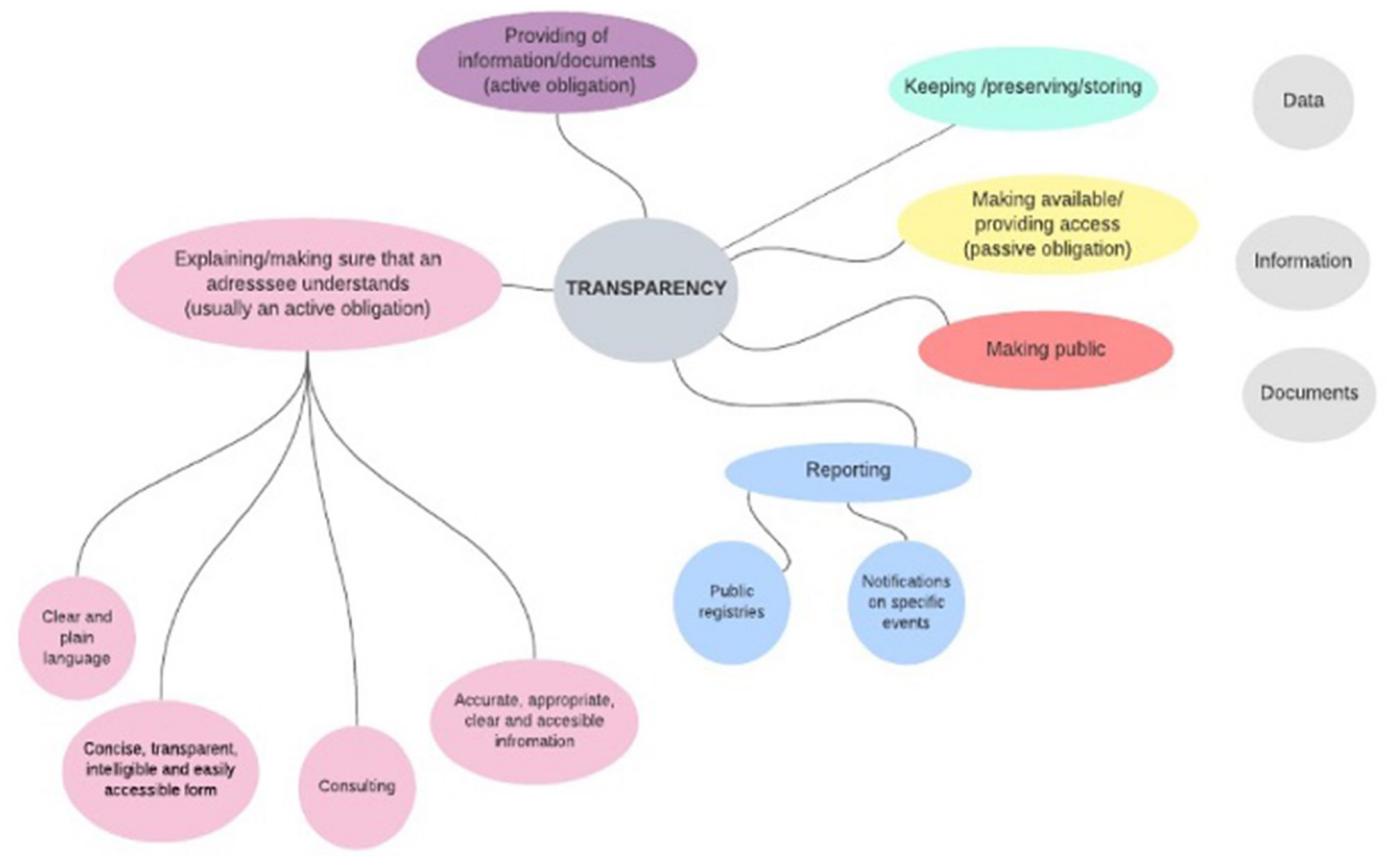
Activities associated in the EU legislation (listed in Annex I) with transparency measures.

At the level of the secondary EU legislation, we analyzed 10 laws from various areas starting from the functioning of financial markets to establishing proper working conditions in the EU (the list of the analyzed sources is provided in Annex I). All of them establish requirements of transparency but none provides its definition. However, these laws refer to certain types of actions when they set transparency rules. These actions concern three main types of objects (data, information, and documents)^9^ and can be divided into a group of active obligations (such as the provision of the mentioned objects) and a group of passive obligations (such as giving access to data/information/documents). Active obligations include explaining and making sure that the addressee understands the provided information; reporting to public authorities and provision of information to public registries; keeping, preserving, and storing. Passive obligations include, for example, making something public. Some of the actions are performed inside an organization (such as preserving and storing) and some actions are addressed outside of an organization (such as reporting and public registries). The graphical summary of the analysis is presented in Figure 1.

Nowadays, discussions about AI's transparency concentrate on solving its opacity issue through interpretability and explainability. Scholars already provide a detailed and useful analysis of legal requirements on explainability in machine learning (Hacker et al., 2020; Bibal et al., 2021). However, our legal picture of transparency (before AI came into play and posed new challenges) demonstrates that transparency is not only explaining something to someone but also concerns varied activities at all the stages of the data/information life cycle. This approach to transparency can be also observed in the policy documents devoted to the development of the AI legal framework.

#### Transparency in the EU Policy Documents on AI

We analyze four policy documents at the EU level on the regulation of AI: AI HLEG Ethics Guidelines (AI HLEG, 2019), the White Paper on AI (European Commission, 2020), the EP Report on AI Framework (European Parliament, 2020), and the EC Proposal for the AI Act (European Commission, 2021). The list of concepts covered by the term “transparency” (or closely related to it) in these documents is rather consistent and includes the following: explainability, interpretability, communication, auditability, traceability, information provision, keeping the records and data, and documentation. However, the policy documents are not consistent in establishing the correlations between all the concepts. Some of them mention transparency as the widest concept (White Paper on AI, 2020, p. 15), whereas others put explainability and transparency at the same level (EP Report on AI Framework, 2021, p. 6).

The latest and the most important policy document (because it is the official legislative proposal) – the EC Proposal for the AI Act – is the least consistent in building the system of transparency-related concepts. First, “the act has two different types of transparency for different types of AI technologies (interpretability for high-risk AI systems (art. 13.1) and communication for interacting AI systems (art. 52.1)” (Kiseleva, 2021a). This means that one term refers to different concepts in different situations which makes compliance with the requirements and exchange of information about it more complicated. Second, unlike the three previous policy documents that explicitly included information provision, records keeping, and documentation in the transparency requirement, the proposed AI Act establishes these requirements in a more detailed way but more separate from transparency (Kiseleva, 2021a). We argue that the mentioned concepts are closely connected to transparency and shall be included in transparency-related measures.

#### Transparency of AI Shall Be Linked to Its Neighboring Concepts

Information is the fuel that enables transparency to exist. The proposed AI Act suggest a very detailed requirement of information provision: it specifies the type of information (AI system's accuracy, performance, intended purpose, foreseeable risks, identity of providers, and its representative), form (concise, complete, correct, and clear information that is relevant, accessible, and comprehensible), its addressees (users of AI systems), and the subjects who shall give the information to users (AI providers) (EC Proposal for the AI Act, art. 13). Importantly, as part of information provision, the AI Act adds a new obligation: provision of instructions to use AI systems (EC Proposal for the AI Act, art. 13). The instructions shall include the specifications for the input data, performance of the system and its predetermined changes, and the intended purpose of the AI system. All these requirements are crucial and help to ensure the overall safety and quality of AI systems. We fully support the suggested requirement but argue that its connection to transparency shall be established in the legislation. Knowing how AI systems shall function, their risks, accuracy, and performance clearly increase their transparency for users.

Similarly, important for transparency are the requirements of records keeping and documentation. Transparency is needed for *ex-ante* and *ex-post* controls ensuring that AI technologies are safe, accurate, and respect fundamental rights. Keeping the records and documentation of all the steps taken during AI's development and use is a good way to organize such control and thus to ensure transparency.

#### Transparency of AI Shall Be Viewed as a Legal Principle and a “Way of Thinking”

Transparency of AI needs to be considered an overarching concept (legal principle). Other concepts (information provision, records keeping and documentation, auditability, traceability, explainability, interpretability) shall be considered the measures to achieve the said principle. This approach already exists in the current legislation, and we take the GDPR (Regulation, 2016) as an example to better illustrate it.

In the GDPR, transparency is established as one of the core principles of data processing – it shall be transparent toward a data subject [GDPR, art. 5(1)]. Many requirements established in the GDPR are aimed to achieve this principle: requirements of information provision and communication imposed to data controllers (GDPR, art. 12-15), obligation to provide access to personal data upon request of data subject (GDPR, art. 15), records of processing activities (GDPR, art. 30), provision of explanation of the decision based solely on automated processing and producing substantial effects on data subjects (GDPR, recital 71), and informed consent requirement (GDPR, art. 7). This makes reaching easier a rather complex principle of transparency through the understanding of what kind of requirements correspond to it and what are the specific measures expected from data controllers. A similar approach shall be taken in the AI Act.

In the future AI legal framework, transparency shall become a “way of thinking” built in the whole life cycle of AI development, use, and control rather than just a single activity to tick the box of the legal obligation. This broad approach helps to set the extended list of measures available to AI providers and users to ensure transparency and comply with the legal requirements. This list of measures is now rather limited due to the technical difficulties with solving AI's opacity and reaching explainability and interpretability. These two concepts and their relevance to AI's transparency are explored in the next section from the point of view of computer scientists.

### Views on AI's Transparency: Data Science

#### The Need for AI's Transparency Taxonomy

**Figure 2:**
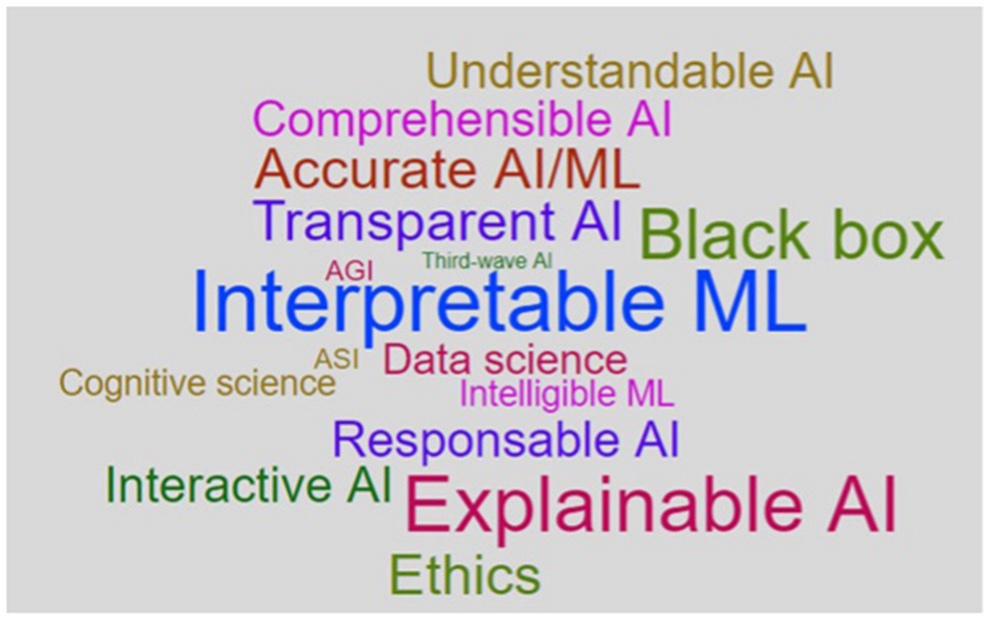
XAI Word Cloud created by Adadi and Berrada (2018).

Transparency of AI in computer science is a rather opaque concept. Despite the substantial amount of research devoted to solving the black-box issue of AI, many scholars in the field agree that a clear relevant taxonomy is missing (Doshi-Velez and Kim, 2017; Adadi and Berrada, 2018; Linardatos et al., 2021). Several terms are used to address the problems of AI's opacity: transparency, interpretability, explainability, comprehensibility, understandability. A. Adadi and M. Berrada explored the concept of XAI (explainable AI) and conducted a linguistic search to identify and record relevant terms across research communities that strongly relate to the mentioned concept (Adadi and Berrada, 2018). The results of their search (presented in Figure 2) illustrate that XAI is not a monolithic concept (and the same can be said about all the terms included in the search such as transparency and interpretability). The table demonstrates that the two most popular concepts are “interpretability” and “explainability” and the first one is used more in the data science community than the second one (Adadi and Berrada, 2018).

Yet, how the concepts of “interpretability” and “explainability” shall be distinguished one from another is an unresolved question. To answer it, several approaches were developed. Some scholars use the concepts interchangeably (Molnar, 2022), others view interpretability as the broadest term that includes explainability measures (Lipton, 2016; Doshi-Velez and Kim, 2017), finally, the third group of scholars takes the opposite approach and operates the term XAI as the main one and includes model's interpretability as one of the sub-categories (Adadi and Berrada, 2018; Linardatos et al., 2021).

Despite different uses of the concepts, many scholars in data science agree on the need for the common taxonomy (Doshi-Velez and Kim, 2017; Adadi and Berrada, 2018). F. Doshi-Velez and B. Kim noted that creating a shared language is essential for evaluation, citation, and sharing of results (Doshi-Velez and Kim, 2017). A. Adadi and M. Berrada stated that “a consensus on definitions must be done in order to enable easier transfer of results and information” (Adadi and Berrada, 2018). We fully support this argumentation and submit that the first step to build the taxonomy is to differentiate between the concepts of “interpretability” and “explainability.”

#### Differentiating Between Explanability and Interpretability

In this paper, we follow scholars who view “interpretability” as the broader concept that, *inter alia*, includes “explainability.” Specifically, we agree with the statement of M. Honneger that “interpretability is the end-goal we want to achieve, and explanations are the tools to reach interpretability” (Honegger, 2018, citation in Carvalho et al., 2019). To support this approach, we see several justifications. First, as discovered by A. Adadi and M. Berrada in their linguistic search, the term “interpretability” is applied more often in the computer science community. Thus, its prevalence over explainability would require fewer adjustments in the future. In addition, in legislation and policy papers, interpretability is something that by default is associated with AI because the term was not in use before the ethical and legal discussions around the “black-box” issue started (unlike explainability that was used before in different domains). Thus, it would make the term “interpretability” more automatically associated with AI and distinguish it from similar concepts in other domains. Second, based on the simplest linguistic analysis, the actions that correspond to the concepts of ‘interpretability’ and ‘explainability’ refer to actions of different types carried out by opposite actors. Explainability is being able to explain something to someone, meaning that it concerns the ability of subjects to generate and deliver explanations. Interpretability, on the other hand, is being able to interpret something. To interpret has several meanings, but in many cases, it concentrates around the ability to comprehend or understand something by the actor. Thus, we need the explanations of one actor for interpretation by the other one.

Explanations might include different measures, both of technical and non-technical natures. Technical measures especially concern the group of *post hoc* explanations – the ones that are applied to explain the decisions of a model after it has been built and used to compute these decisions (predictions) (Honegger, 2018). This type of measures does not require to alter or understand the inner workings of algorithmic models (Adadi and Berrada, 2018) – in other words, to open a black-box. Currently, a substantial amount of research in computer science focuses on developing automatically generated explanations – a new generation of AI technologies called the third wave AI where one of the objectives is to precisely generate algorithms that can explain themselves (Adadi and Berrada, 2018). Non-technical explanations refer to oral or written clarifications about AI, its functioning, and other important parameters. In healthcare, it can concern clarifications provided by healthcare professionals to patients when informing them about a treatment with the use of AI systems or clarifications given by AI developers to healthcare providers. The human model explanations such as causability can be also included in non-technical explanations (Holzinger et al., 2017; Holzinger and Mueller, 2021)^10^. In this paper by explanations, we refer to all its types, technical and non-technical.

As mentioned, interpretability is something that we want to reach through explanations. In turn, defining and evaluating interpretability should be context dependent (Doshi-Velez and Kim, 2017). To understand if we reached interpretability, we have to define who is the actor of interpretability (who tries to comprehend) and why it is needed. The answers to these questions are always based on the context. In healthcare, the interpreting actors are AI providers, AI users (healthcare professionals), and AI beneficiaries (patients). They need to interpret the AI systems and their outcomes to make informed decisions related to their roles in the process of AI's development and use^11^.

#### Transparency as a Wider Concept That Includes Interpretability and Explainability

After differentiating “interpretability” from “explainability,” the two concepts shall be correlated to transparency. Again, there is no consensus on it among computer scientists. Some of them understand transparency as a “human-level understanding of the inner workings of the model” (Belle and Papantonis, 2021, with reference to Lipton, 2016) and see transparency as part of interpretability (Lipton, 2016) or explainability (Adadi and Berrada, 2018). As mentioned by Z. Lipton, “transparency is the opposite of opacity or black-box-ness” (Lipton, 2016). Yet, other researchers view transparency as a wider concept and describe it as “a need to describe, inspect and reproduce the mechanisms through which AI systems make decisions and learn to adapt to its environment and to the governance of data used to create it” (Adadi and Berrada, 2018). We support this approach due to several reasons. First, unlike “interpretability” and “explainability” that refer to the actions of humans (or their abilities), “transparency” characterizes objects or processes. Thus, it is the concept that can be applicable to the whole process of AI's development and use, to its life cycle, and cover different measures, not only to the ability of human actors to comprehend AI and its outcomes. Second, the broader vision on transparency is aligned to how it is perceived in legal scholarship and thus works better to develop and use the common interdisciplinary terminology. This type of terminology is the first and necessary step to the approach toward reaching the AI's transparency. In this sense, transparency is the overarching value and characteristic of the whole process to develop and use AI, affecting all its elements and actors. The vision on transparency common for law and data science is summarized in the following subsection.

### Summary and Suggested Vision

#### The Need to Distinguish Between Transparency of Algorithms and Transparency of the Use of Algorithms

The previous two sections demonstrated that both in law and computer science, transparency is the concept that is not yet cast in stone. While the AI Act is in its preparatory process, the sooner we start developing the interdisciplinary approach to create the common transparency taxonomy, the higher chances that the adopted legislative act reflects the vision shared between practitioners and scholars coming from different disciplines.

The EP Report on AI Framework noted the “important distinction between transparency of algorithms and transparency of the use of algorithms” (European Parliament, 2020, recital 20). This distinction is crucial for this work. It is the algorithm that causes technical issues with explaining decisions made with the use of AI. But everything other than the algorithm does not have this issue. It concerns both the other element of AI system (data) and human actions related to the development, deployment, and use of AI systems. It means that transparency measures should also focus on controllable elements of the AI systems: data governance and management^12^, roles, and responsibilities of humans (including their organizations) involved in the AI lifecycle. This approach does not exclude the necessity to solve the technical issues related to the opacity of AI systems, but it allows not to limit the transparency measures to only technical ones and to only explainability and interpretability.

#### Transparency as an Umbrella Concept Achieved Through the Set of Technical and Non-technical Measures

We argue that transparency shall be viewed as the broadest concept, as a continuous process accompanying the whole life cycle of AI system, and as a legal principle. As the principle, transparency shall be achieved through the set of measures established in law. These measures shall include the ones that concern the black-box issue of AI (interpretability and explainability) and the ones that concern the observability (Rieder and Hofmann, 2020) and control over the whole process of AI's development and use (communication, auditability, traceability, information provision, keeping the records, data governance and management, documentation).

To reach a common terminology, the AI's interpretability and explainability shall be distinguished. We suggest that explainability shall refer to the ability to *explain something* (*to provide* explanations), while interpretability is the ability to comprehend *something*. It means that we achieve interpretability as the end goal through explanations, and we need explanations of one actor for interpretation by the other one. In this system, transparency characterizes the whole process of AI's development and use, and interpretability characterizes the human perception of AI system and its outcomes.

However, the implementation of a transparency system is only possible when it is contextualized. Scholars in both computer science (Lipton, 2016; Doshi-Velez and Kim, 2017; Rudin, 2018; Costabello et al., 2019; Ghassemi et al., 2021; Holzinger and Mueller, 2021) and law (Felzmann et al., 2020; Astromské et al., 2021) agree that transparency measures should depend on the area of AI application. Placing AI's transparency in a specific context enables to take into consideration the existing relevant legal frameworks that establish rights and obligations for involved subjects, including those related to transparency. In addition, the transparency's contextualization allows to formulate its functions in the specific field and thus to develop guidance on the measures applicable to AI. The next section explores this topic to further define the applicable legal frameworks.

## Functions and Types of AI's Transparency in Healthcare

### Functions of AI's Transparency in Healthcare

To better define transparency and to shape the measures to achieve it, it is first necessary to answer the question of *why* transparency is needed. The answer depends on the domain where this question is posed. In this paper, the domain is the use of AI in healthcare.

Literature on healthcare transparency identifies various functions it can serve. Nicolaus Henke, Tim Kelsey, and Helen Whately suggested six benefits of transparency in healthcare: accountability, choice, productivity, care quality/clinical outcomes, social innovation, and economic growth (Henke et al., 2011). Computer scientists identify the following functions of AI's explainability: to justify (to see if and why the AI's decisions are erroneous), to control (to prevent things from going wrong), to improve (knowing how the AI system reached the specific output enables making it smarter), and to discover (asking for explanations is a helpful tool to gain knowledge) (Adadi and Berrada, 2018). These functions also correlate with the ones identified in legislation and in legal literature: accountability (Felzmann et al., 2020; Rieder and Hofmann, 2020); ensuring efficacy, safety, and quality (Carvalho et al., 2019); improving trust (Kiseleva, 2020a); making informed decisions [CFR (Charter of Fundamental Rights of the European Union, 2012, art. 3) and Oviedo Convention (Convention for the Protection of Human Rights, 1997, art. 5)]; realization of individual rights [CFR (Charter of Fundamental Rights of the European Union, 2012, art. 3) and Oviedo Convention (Convention for the Protection of Human Rights, 1997, art. 5)].

In this paper, we propose these functions of transparency:

1) accountability (that, *inter alia*, covers control and justification);
2) ensuring safety and quality of AI in healthcare (which also enables improving AI);
3) making informed decisions (which in the case of patients also leads to the realization of individual rights).

These functions are specific enough for shaping the system of transparency that concerns all the involved subjects, including their rights and obligations. While we admit the importance of other functions (such as trust, social innovation, and economic growth), we do not focus on them because they are difficult to measure and can be described as secondary or indirect positive effects of transparency.

The first domain where transparency proves its utility is accountability. “Accountability can be considered the alter ego of transparency (De Hert, 2017, with reference to Tzanakopoulos, 2014 and Harlow, 2002), and at the same time the final good to which transparency is instrumental” (De Hert, 2017). As with transparency, there is no universally accepted definition of accountability (De Hert, 2017). According to D. Brinkerhoff, “being accountable means having the obligation to answer questions regarding decisions and/or actions” (Brinkerhoff, 2004). We would suggest that besides the obligation, accountability also refers to the capability to answer and this is where transparency is especially important. “Transparency enables the relevant subjects to explain their actions and to provide the required information necessary for justification and assessing their performance” (Kiseleva, 2020a; Rieder and Hofmann, 2020)^13^. Transparency can be a “powerful driver of accountability” for different stakeholders such as health regulators, healthcare providers, and patients. Thus, transparency is essential to ensure accountability.

Increased accountability and advanced patients' choices are closely linked to another crucial function of transparency in healthcare – *guaranteeing quality and safety*. Generally, in healthcare, ensuring the safety and quality of medical services is crucial because stakes are really high – the health and/or life of an individual. In turn, it is only possible through continuous collecting, generating, and verification of data, information, and knowledge^14^. In the AI context, transparency for ensuring safety and quality is even more crucial. It is needed for both *ex-ante* and *ex-post* control over the AI's outcomes. The accuracy of AI's outcomes depends on the quality and relevance of the inputs and thus the relevant procedures to verify it (control over training and validating data) have to be established. At the same time, the mechanisms to assess the specific output when an AI system is used in real life shall be developed, too. This assessment is possible not only through explanations but also through keeping the records of AI's development and testing, tracing the steps of this process, and establishing procedures of data governance and management. ‘An interpretation for an erroneous prediction helps to understand the cause of the error’ (Carvalho et al., 2019). Transparency enables AI systems to be tested, audited, and debugged, which is a path toward increasing their safety (Carvalho et al., 2019)^15^.

A third goal that transparency helps to achieve is making informed decisions, which concerns different subjects. For patients, it is closely linked to their empowerment and to the requirement of informed medical consent. This right, in turn, relates to the protection of human dignity.^16^ To make decisions about their health, patients shall have access to information about their health conditions, diseases, risks and outcomes of suggested treatment options, their costs, and alternatives. More importantly, they shall fully understand the provided information.

Healthcare professionals shall also be provided with the tools to make their informed decisions. Communicating with patients, physicians shall make sure that their explanation is sufficient for making informed choices. However, in the AI context, the ability to comply with the requirement of informed consent depends not only on the medical knowledge of healthcare providers (as is normally the case) but also on their comprehension of AI-based devices and their decisions. In addition, physicians shall be provided with the information that enables them to choose in what situations to apply AI tools, how to use them, and how to verify the results that an AI system suggests.

### Types of Transparency in Healthcare

The functions of transparency identified in the previous section (accountability; choice; safety, and quality) concentrate on the roles of the concerned subjects, their rights, and obligations. To continue building the system of transparency in this direction, we classify transparency also based on the stakeholders' roles: who shall be transparent to whom. This system is identified from the literature on healthcare transparency and is further developed to tailor it to applying AI in the domain (Kiseleva, 2020a).

Gary S. Kaplan suggests the following categorization: external and internal types of healthcare transparency. According to him, transparency of healthcare providers to patients implicitly refers to “external transparency” (Kaplan, 2018). Internal transparency is the transparency “among all team members, at all levels, on all issues — throughout the health care organization itself” (Kaplan, 2018). Supporting the offered approach and taking it as a basis for further analysis of AI's transparency, in this article, we view the internal transparency broader than transparency inside a healthcare organization. It is suggested that internal transparency shall refer to all the stakeholders (except for patients) directly or indirectly involved in healthcare provision and influencing the possibility to sufficiently provide external transparency (depending on its specific focus). In this case, the public authorities, manufacturers of medical devices, pharmaceutical companies, and other similar subjects shall be considered at the level of internal healthcare transparency^17^.

In the development of the classification suggested by Gary S. Kaplan, we propose distinguishing as part of internal transparency the so-called “insider” transparency. This type of transparency is necessary for the AI context to separate the roles of AI providers who are considered insiders and the roles of healthcare professionals who are considered the subjects of internal transparency. Insider transparency is addressed from AI providers to themselves and refers to their ability to comprehend the AI system and its decisions. Internal transparency is addressed from AI providers to AI users – healthcare professionals. Finally, external transparency refers to the relationship between healthcare providers and patients.

This classification of transparency is the basis for further analysis in this paper. We explore the legal frameworks applicable for use of AI in healthcare in relation to the type of transparency they correspond to. For external transparency, we examine the requirement of medical informed consent as the one that ensures the informed choice of patients and realization of their right to dignity. For internal (including insider transparency), we analyze the rules of the MDF that govern the verification of AI-based medical devices, both before and after they are placed on the market. In this structure of the analysis, the functions of transparency identified above (accountability, choice, safety, and quality) are also organically implemented in the frameworks.

### Transparency, Accountability, and Risk Management

#### Accountability as a Methodology to Build AI's Transparency System

One of the main functions of transparency is to ensure the accountability of all the involved subjects^18^. Transparency is needed for them to justify their actions and to organize control over their activities. However, in the AI context, the reverse correlation between accountability and transparency becomes especially important. The black-box issue leads to limitations in interpreting the outcomes of AI systems and, because of that, building a strong accountability system is one of the ways to ensure transparency^19^. When all the concerned subjects can justify their actions and ensure the proper control, any process inherently becomes more transparent.

The multilayered system of transparency^20^ shall establish the rights and obligations of the concerned subjects by defining addressees and addressors (who to whom), objects (what), and timing of transparency (when). These factors together with measures (how) shall be guided by the functions of transparency (why)^21^. This system is based on accountability tools of different natures: external (to set the relevant structures that enable control), internal (duty to justify your actions and develop practices for that), and self-imposed (De Hert, 2017).

**Figure 3:**
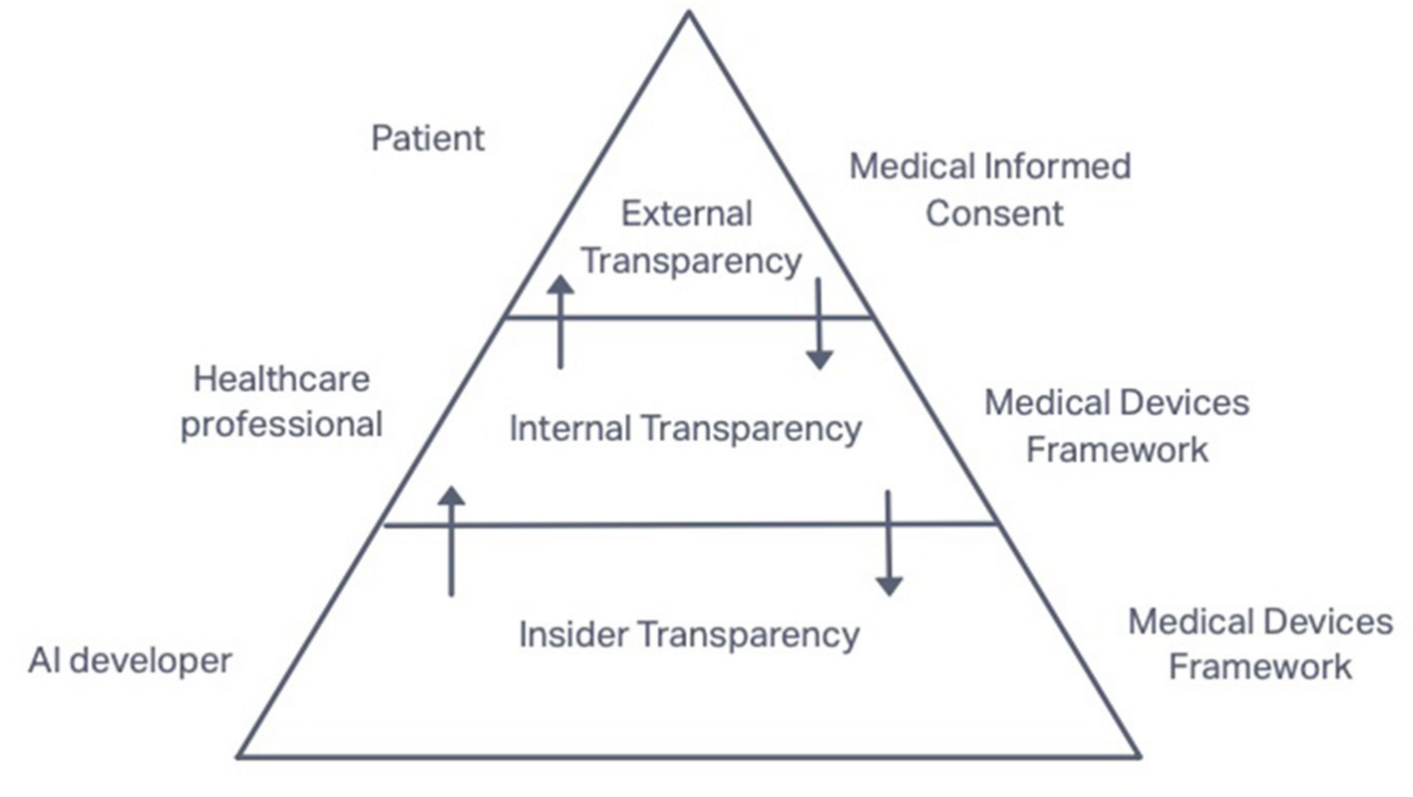
Multilayered System of AI's Transparency in Healthcare.

All the elements and layers of the transparency system are interrelated and can be described as a ripple effect. External transparency (toward a patient) is not possible without the internal one (toward a healthcare provider), which, in turn, is impossible without the insider transparency (toward an AI provider). At the same time, the needs of patients shape the obligations of a healthcare provider, and his needs shape the obligations of AI providers. However, at every layer, the transparency measures will vary depending on the context, knowledge of the addressee, choices that transparency helps to make to the addressee, and generally the positions of addressor and addressee in the whole process (and their obligations)^22^. Yet, the measures at one level influence the measures on other levels and vice versa. The picture of transparency layers together with the corresponding legal frameworks and main actors is represented in Figure 3.

The multilayered transparency system based on accountability tools also enables to implement transparency measures to already existing legal frameworks. The use of AI in healthcare does not arrive as a rocket to outer space, it is landed to the existing compliance procedures such as verification and authorization of medical devices that guarantee their safety and quality^23^. The need to implement the future AI framework (including the requirement of AI's transparency) into existing procedures and rules is also expressed by the EU legislator. The proposed AI Act states that the requirements to high-risk AI systems shall apply to AI devices covered by the MDF. The two frameworks will work together: “the safety risks specific to AI systems are meant to be covered by the requirements of the AI Regulation, and the sectorial legislation (MDF) aims to ensure the overall safety of the final product” [EC Proposal for the AI Act (European Commission, 2021, p. 4)].

#### Implementation of Existing Risk-Management Approach in Healthcare to AI's Transparency

The implementation of AI-related transparency measures into existing legal frameworks leads to the final point of this section: the need to take into consideration the risk management systems established by these frameworks. The approach to this type of systems suggested in the proposed AI Act is rather one sided because it considers only possible risks and their prevention (European Commission, 2021, art. 9). In contrast, the MDF allows to admit some risks acceptable if they are overweighted by the relevant benefits [MDR (Regulation, 2017a, Annex 1, Chapter 1, art. 4)]. Moreover, the framework requires minimizing the risks to the only extent that does not adversely affect the benefit-risk ratio (MDR, Annex 1, Chapter 1, art. 2). In AI context, the perspective to look at risks in comparison to the relevant benefits is important, especially concerning the trade-off between AI's accuracy and explainability^24^. Risks must be reduced to the possible minimum level, but it is crucial to realize that zero risks are impossible in any area. Of course, in healthcare, the risks are of very high stakes because they concern “physical harms—death, disability, disease that rob us of normal and foundational powers of action” (Keating, 2018). However, even in this case, ‘the risks in question should be reduced as far as possible without “killing the activity” in question’ (Keating, 2018). Gregory C. Keating mentioned:

‘*A world of “no risk” is not a world worth having. Diminished security is the byproduct of action. Diminished liberty is the price of increased security. We cannot farm, build, drive, fly, eat and drink, or mill cotton and refine benzene without taking and imposing risks of devastating injury. A world in which no one moves is a world in which few, if any, aims, ends, and aspirations can be realized, and few, if any, lives can be led’ (Keating*, *2018**)*.

Besides the need to accept some risks in consideration to the relevant benefits, one shall bear in mind that risks can be unpredictable, especially concerning health. “Medical treatment itself is always an unpredictable and risky process and there is no universal standard of treatment that suits everyone” (Kiseleva, 2020a). “Each man and woman is ill in his or her own way” (Topol, 2019). Human bodies are unique and can respond differently to a treatment. In other words, no one can predict the result of suggested treatment with 100% definitiveness' (Kiseleva, 2020a). In the end, risks can never be minimized to a zero level.

We argue that a future framework on AI, including its transparency requirements, shall be based on the approach that not only tries to minimize risks to the maximum possible extent but also views them in consideration to relevant benefits. Healthcare legislation (MDF) already follows this approach and allows to accept some risks despite high level and sensitive nature of risks in this area. Both the inherent “black-box” issue and lack of full predictability (due to constant self-learning) of some AI applications shall be analyzed as the risks that might be acceptable when the benefits of AI's use overweight them and where are yet no technical solutions to solve the said risks^25^. The two legal frameworks applicable to the use of AI in healthcare are analyzed further in the light of this approach. The analysis starts with the informed consent requirement.

## External Transparency of AI in Healthcare: Toward Patients

### Rule of Informed Medical Consent as the Foundation for External AI's Transparency in Healthcare

In this section, we explore the external transparency of AI in healthcare – the one that is addressed toward the recipients of medical care (patients)^26^. The legal framework that corresponds to this type of transparency is the requirement of medical informed consent. The general requirement is established by the international treaties – Oviedo Convention (Convention for the Protection of Human Rights, 1997, art. 5) and the EU CFR [Charter of Fundamental Rights of the European Union, 2012, art. 3.2(a)]– and detailed by national legislations. Although states can establish their own nuances in the rule of informed consent, the basis of the requirement is expressed in the Oviedo Convention, and in the interest of generalization and uniformity, we take this document for the analysis in this paper.

Article 5 of the Oviedo Convention states: “An intervention in the health field may only be carried out after the person concerned has given free and informed consent to it. This person shall beforehand be given appropriate information as to the purpose and nature of the intervention as well as on its consequences and risks” (Convention for the Protection of Human Rights, 1997, art. 5). This legal requirement already enables to define some of the transparency elements (partly or fully) to be constructed in this paper by the accountability approach. These elements are represented in Table 1.

**Table 1:** Summary of the requirement of the informed medical consent applicable to artificial intelligence (AI)'s external transparency.

Who to whom:	Healthcare professional (physician) to patient^a^;
When:	Before intervention in the health;
What:	Information as to the purpose and nature of the intervention as well as on its consequences and risks;
How:	Appropriate information;
Why:	To enable free and informed consent/rejection of the intervention.

a *Although the article does not directly mention healthcare professional as the one required to provide information to patient, it is implied because he is the one who carries out intervention into health under its professional duties*.

Table 1 demonstrates that at least two elements of the external transparency layer are clearly defined by the corresponding legal framework (who to whom and when). Other elements (what, how and why) are described just partly, and in this section, we aim to provide some guidance for their specification in the context of the use of AI for diagnosis and treatment.

### Justifications of an External Transparency of AI in Healthcare

To answer the questions of how and what, we first have to understand **why** transparency is needed. At the level of transparency toward patients, its main function under the legislation is enabling patients' choice on interference in their health which refers more fundamentally to their mental and physical integrity. The ability of patients to make choices expresses the movement toward their empowerment that started in the second half of the 20th century and replaced the prevailing before “doctor knows best” paternalistic approach (Verhenneman, 2020, p. 7). Accountability is another function that transparency helps to reach at this layer because the obligation to provide information about the suggested treatment and its alternatives, risks, and consequences makes physicians justify their actions and decisions and answer to patients about them. Finally, indirectly, it all can increase the safety and quality of medical services because shared decision-making can raise self-awareness and self-care of patients. It can motivate them to provide more information to healthcare providers which, in turn, can reduce medical errors or suffering from health consequences caused by miscommunication.

### Measures of External Healthcare Transparency in AI Context

Based on the defined functions of external transparency, we now need to answer the question of external transparency's scope and measures in AI context^27^. The measures to achieve AI's transparency toward patients are^28^ interpretability and explainability, communication, and information provision. These measures also shape the scope of transparency.

**Communication (measure 1)** means notifying patients that AI device is used in their diagnosis and treatment. Although, as identified by I. Glenn Cohen, it is not fully clear if the current legislation, in fact, establishes this obligation (Glenn Cohen, 2020), we argue that it shall be the necessary element of the transparency process. The patient shall have the right to choose whether to be diagnosed and treated with AI facilities or not; otherwise, his integrity can be violated.

**Provision of information (measure 2)** is directly specified in the requirement of informed consent. We argue that in the AI context, this provided information shall include the fact of the usage of AI facilities (as the communication measure) and possible alternatives to it (if and how the suggested diagnosis/treatment can be reached without AI). The patient shall be also informed on how is substantial the role of the decision made with the use of AI in the whole treatment process (compare, for example, AI-based analysis of medical image and AI-based decision on the need for surgery for the specific patient – the importance of AI's use is different in these situations). A physician shall also notify a patient on the fact that AI was certified under the conformity assessment procedures and its safety and quality were verified before the AI-device's application within the healthcare organization. In addition, healthcare professionals shall inform patients on the intended purpose of the AI system and its level of accuracy demonstrated during the market authorization. The data about a patient that was added to the AI system as an input for the decision shall be specified and communicated to a patient. All these types of information are feasible to provide even for the opaquest systems, rather comprehensible, and are useful to inform patients on the purpose and partly on the nature, consequences, and risks of AI-related intervention^29^.

Finally, in AI context, the most challenging and debatable transparency measure toward patients is **explainability (measure 3)**, that is needed to reach interpretability. In this regard, we submit several suggestions. First, providing explanations shall be part of external transparency^30^. It can concern any question that a patient can pose about AI's use and shall serve with the aim to answer that question so that the patient understands and can make the relevant choice. These explanations are provided by a physician but can include the ones embedded by design into AI systems by AI developer. This vision is inspired by the concept of AI's transparency by design suggested by legal scholars who view it broadly as a set of measures of different natures and from different actors (Felzmann et al., 2020). We agree with the H. Felzmann et al. that transparency shall be a holistic concept and be considered at the earliest stages of AI development^31^. We suggest the term ‘explanations by design’ to refer to the technical tools embedded in AI's functionality to explain the AI system and its outcomes – in data science, it is also known as “automated explanations.” This concept is narrower than transparency by design and helps to differentiate technical explanations from the other types (for example, explanations provided by physicians)^32^. Explanations by design can be addressed to AI users (healthcare professionals) or the subjects of AI decision-making (patients) or both. This type of tools can explain the general functionality and purpose of the AI system and/or its specific outcome in relation to the specific patient (which is more challenging due to black-box issue). Finally, the embedded explanations can be based on different explanation techniques developed in data science^33^, but have to be adapted to the needs of the audience because the law requires the information to be provided in appropriate form^34^. For patients, it could be videos and/or images accompanied with the text in plain and accessible language.

### Suggested Considerations for AI's External Transparency in Healthcare

The most challenging question is how to provide explanations to the systems that are opaque even for their developers. In this case, we argue that the best explanations possible shall be provided, but some level of opacity shall be considered one of the acceptable options. Following the risk-benefit approach discussed earlier, the inherent opacity of some algorithms shall be assessed during the verification of a AI-based device toward their performance^35^ and other benefits on their own and in comparison with the existing alternatives. Included in the broad set of transparency measures, all the possible AI's explainability measures (technical and non-technical) to achieve interpretability shall be taken, but the inherent opacity of AI algorithms can be accepted as one of the AI's risks. In this situation, the importance of ensuring the accuracy of AI's decisions becomes even stronger.

In the context of AI's transparency toward patients, we have to keep in mind that not everything has to be explained. Too much information can make the decision-making more complex and thus prevent from reaching the main goal of transparency – enabling making choice. “Many AI technologies are complex, and the complexity might frustrate both the explainer and the person receiving the explanation” (WHO Guidance, 2021). In addition, many believe “that ‘overdisclosure’ makes it difficult for patients to distinguish the meaningful risks from trivial ones” (Glenn Cohen, 2020). “Explainable machine learning is not dissimilar from how a clinician must distill extremely complex reasoning based on decades of medical education and clinical experience into a plain-language explanation that is understandable to a patient” (Petch et al., 2021). Medical processes with and without the use of AI already reach a complexity today that obliges medical personnel to explain with “appropriately reduced complexity” (Schneeberger et al., 2020 with reference to Eberbach, 2019). Consider the example with prescribing medicines – a physician does not explain to a patient the chemical formulas inside the medicine because this would not help a patient to decide about taking it (Kiseleva, 2020a). Instead, a patient is informed on why a physician prescribed the specific medicine, about its side effects and risks, as well as on benefits of taking the medicine (Kiseleva, 2020a). At the same time, another analogy can be used – when a physician makes any treatment or diagnosis decision, we are not exploring how his brain is functioning for that specific decision^36^. It is not needed and is not possible yet. Z. Lipton mentioned the black-boxness of the human brain (Lipton, 2016) and, indeed, how exactly our brain works is one of the unresolved mysteries in science. Yet, it does not prevent us to act in complex and in different environments and justify our actions (including through different explanations).

Finally, we argue that AI's external transparency shall be always tailored to the needs of the specific patients (Mourbya et al., 2021), and this tailoring is always conducted by a healthcare professional. A physician is the one who makes the suggested medical decisions and the one who shall explain them to a patient in an appropriate-for-that-patient manner. He is the one who directly interacts with a patient and can assess his knowledge and their motivation in the decision-making process. “The process should be individualized within the boundaries of the patient's desires for autonomy, thus reflecting true patient autonomy” (Paterick et al., 2008). The same applies to the explanations about AI algorithms, their inherent opacity, and risks about changing the accuracy of AI algorithms due to self-learning (risks of mismatch) (Kiener, 2021). M. Kiener argued that these so-called meta-risks (a risk that the risk-assessment is wrong) are normally not required to be disclosed, but in AI context, the disclosure decision shall be “personalized, i.e., specifically tailored to a patient's personal characteristics” (Kiener, 2021). Generally, although the minimum standards of transparency shall be established and, in this section, we attempted to suggest them, the details of the transparency process toward a patient will be always tailored by a physician^37^. However, in the AI context, the external transparency is highly dependent on the internal one which is discussed in the next section.

## Internal Transparency of AI in Healthcare: Toward Healthcare Providers

### Justifications of AI's Internal Transparency

In this section, we explore the AI's internal transparency – the one that is addressed toward the users of AI devices. In the healthcare context, users are usually represented by physicians or other medical professionals (for example, radiologists, surgeons). Because their role is at the medium level of the transparency system, their relevant rights and obligations are guided by several frameworks. To structure the analysis of these frameworks, we first identify **why** AI's transparency is needed for healthcare professionals.

Internal transparency of AI is initially needed to hold healthcare professionals accountable toward patients and to help the patients in making their treatment choices. In this case, their transparency obligations (toward patients) and rights (toward AI developers) are guided by the framework on the medical informed consent^38^. In addition, physicians have to make their own choices about the use of AI device with regards to the specific patient. This choice concerns both the need to use AI in a specific case and the accuracy of the decision in that case. While physicians are the subjects who are legally responsible for the quality of medical care and can be held liable for medical errors, they have to make sure that the outcome of an AI system is safe. Before starting using an AI device, they have to be not only convinced that the specific outcome is safe, but also the AI device is generally useful, safe, and efficient. For that, they need to have all the necessary information, documents, and explanations provided directly (through interaction and cooperation) or indirectly (through the process of AI's device verification and authorization) by AI developers. The main framework that establishes the rights and obligations of AI providers during the whole life cycle of AI device is the MDF and it is explored here on its ability to perform the functions of the AI's internal transparency.

### Medical Devices Framework as the Foundation for AI's Internal Transparency

The main scope of the MDF^39^ is to ensure the safety and quality of medical devices before and after they are placed on the EU market (MDR, recital 2). For that, the framework establishes the obligations of devices' manufacturers (AI providers in the context of this paper). The importance of transparency and its contribution to the functions identified above is recognized in the law. The MDR in several recitals establishes that provisions ensuring transparency are needed to improve health and safety (MDR, recital 4), as well as for empowering patients and healthcare professionals and enabling them to make informed choices (MDR, recital 43). Although the framework does not establish transparency as a principle and does not explicitly classify what kind of measures shall be covered by the concept of transparency, the importance of transparency recognizable by the framework can justify the link between transparency and measures established by the framework that are analyzed below.

The MDR establishes the types of information supplied by device manufacturers together with the device. This includes “information about safety and quality of the devices **relevant to the user**, or any other person, as appropriate” [MDR, Annex I, Chapter III, 23(1)]. Importantly, the framework highlights that the information about the device, including instructions for its use, shall be addressed to the device's intended user and tailored to his “the technical knowledge, experience, education or training” [MDR, Annex I, Chapter III, 23(1)(a)]. “In particular, instructions for use shall be written in terms readily understood by the intended user and, where appropriate, supplemented with drawings and diagrams” [MDR, Annex I, Chapter III, 23.1(a)]. The framework also specifies the type of information to be provided to the users together with the device: clinical benefits to be expected [Annex I, Chapter III, 23.4(c)]; performance characteristics of the device [MDR, Annex I, Chapter III, 23.4(e)]; information allowing the healthcare professional to verify if the device is suitable [MDR, Annex I, Chapter III, 23.4(f)]; any residual risks, contra-indications, and any undesirable side effects, including information to be conveyed to the patient in this regard [MDR, Annex I, Chapter III, 23.4(g)], as well as all the information that allows healthcare professionals and patients to be properly informed on the risks [MDR, Annex I, Chapter III, 23.4(s)]; specifications the user requires to use the device appropriately, e.g., if the device has a measuring function, the degree of accuracy claimed for it [MDR, Annex I, Chapter III, 23.4(h)]; any requirements for special facilities, or special training, or particular qualifications of the device user and/or other persons [MDR, Annex I, Chapter III, 23.4(j)].

A summary of measures already existing in the legislation and relevant to AI's internal transparency is presented in Table 2. This summary is to inform the AI developers on what is already needed from them under the current framework and continue the discussion of what further adaptations are required.

**Table 2:** Summary of the Medical Devices Framework (illustrated by the MDR) requirements relevant to AI' internal transparency measures.

Who to whom:	AI provider to healthcare professional (device's user) and patient;
When:	When a device is placed on the market and being used;
What:	Providing of information about: • Instructions for the appropriate use of a device; • Safety and quality of a device; • Expected clinical benefits; • device's performance characteristics; • Information allowing assessment of the device's suitability; • Residual risks, contra-indications, and any undesirable side-effects; • Information allowing being informed about risks, contra-indication, and undesirable side-effects; • Requirements for special facilities, or special training, or particular qualifications of the device user and/or other persons; • Specifications the user requires to use the device appropriately.
How:	Information relevant to the user and tailored to his technical knowledge, experience, education, or training. Instructions for use shall be written in terms readily understood by the intended user and, where appropriate, supplemented with drawings and diagrams.
Why:	• To enable healthcare providers making choices, including diagnosis and treatment ones; • To hold healthcare providers accountable, including with regards their transparency obligations toward patients; • To ensure safety and quality of AI-devices, both at the general level and with regards to the specific patient.

Table 2 and its analysis illustrate that the MDF is a good basis point for ensuring transparency addressed to AI users (healthcare professionals)^40^. Importantly, the framework requires the provided information to be relevant to physicians and patients to help them make their choices. It enables to facilitate the multilayered system of transparency in the AI context. However, some adaptations (or interpretations) (Lognoul, 2020) to the nature of AI technologies, their opacity, and self-learning shall be made by policy makers for ensuring transparency at the internal level. The suggested adaptations might be implemented in the next version of the AI Act proposal or included in the thematic guidelines to be issued.

### Suggested Adaptations of the MDF to Ensure AI's Internal Transparency

The first adaptation of the MDF to ensure AI's internal transparency concerns information provision. The list of information to be provided to devices' users (healthcare professionals) shall be specified. The first element of the specification is the information about the data. While the quality of data defines the quality of AI's decisions, data governance and management practices shall be developed by AI providers. They have to inform the users on what kind of data was used to train and validate an AI system and how system parameters might change depending on the input data. Users have to be instructed on what input data are considered to be relevant and appropriate for the AI system in question because it is one of the main tools to keep the accuracy of AI system at the level established during the device's verification (the more relevant the data, the more predictable the outcome). Second, the required information about the device's risks, side effects, and limitations shall cover the risk of algorithmic changes, AI providers' predictions of that changes, as well as the explainability limitations when it is applicable (black-box models). For the opaque models, AI providers have to also inform users on why and how the benefits of the use of this system overweigh its risks (level of accuracy, comparison with other technologies, or practices available on the market).

The second needed adaptation of the MDF concerns explainability measures – the ones that help to clarify provided information and thus to reach interpretability by AI's users. The measures shall concern both general explanations about how AI systems function, what are the important factors to consider during its use, and, more specifically, explanations about AI's outcomes reached with regards to a certain patient. AI providers shall develop possible technical measures to implement automatically generated explanations into their AI systems (‘explanations by design’)^41^ and to complement these technical measures with textual and visual explanations.

### The Application-Grounded Evaluations Suggested by F. Doshi-Velez and B. Kim: How to Implement Them to the MDF to Ensure Contextualized Interpretability

As already required under the current legal framework and argued in this paper, all the transparency measures shall be contextualized and tailored to users of AI systems – healthcare professionals. However, data scientists are not necessarily the experts in the domain and thus might have difficulties in deciding what serves as a good explanation for healthcare professionals. For that, we suggest implementing the taxonomy of evaluation approaches for interpretability developed by F. Doshi-Velez and B. Kim. They differentiate the following levels of interpretability evaluations: application grounded, human grounded, and functionally grounded. The one that is relevant at the users' level is the application-grounded evaluation of interpretability.

“Application-grounded evaluation of interpretability involves conducting human experiments within a real application” (Doshi-Velez and Kim, 2017). “If the researcher has a concrete application in mind—such as working with doctors on diagnosing patients with a particular disease—the best way to show that the model works is to evaluate it with respect to the task: doctors performing diagnoses” (Doshi-Velez and Kim, 2017). F. Doshi-Velez and B. Kim argued that the quality of the explanation shall be evaluated in the context of its end task and that an important baseline is how well human-produced explanations assist in other humans trying to complete the task (Doshi-Velez and Kim, 2017)^42^. We support this vision and suggest that this type of interpretability evaluation shall be implemented into the MDF. Specifically, when AI-based device goes through the conformity assessment procedure established by the framework, the quality of explanations developed by the AI provider to be supplied together with the device in question shall be assessed by healthcare professionals specializing in the area of the device's use^43^. This would contextualize explanations not only generally to medical field but also to its specific sub-field. This is because explanations in cardiology, for example, would definitely have some differences from explanations in radiology, as well as the details that are needed by professionals in these areas. To organize this type of healthcare professionals' involvement into the devices' verification process, some sort of independent bodies representing healthcare professionals for their participation in the AI-device evaluation can be created. This would ensure independency of the involved healthcare professionals from AI providers and release AI providers from the need to search for the evaluators of their explanations.

However, the extent of explanations to users depends on how much AI providers can interpret themselves the outcomes of an AI system - they cannot explain more than they understand. Some AI models are opaque even for their creators, and this issue brings us to the last level of transparency – insider transparency.

## Insider Transparency of AI in Healthcare: Toward AI Developers

### Justification of AI' Insider Transparency

We suggest introducing the concept of insider transparency – the one that is addressed to AI providers from themselves. As discussed above, the first transparency measure concerning this type of transparency is explainability. To perform their duties toward the users of AI systems and to provide them contextualized and comprehensible explanations, AI providers shall first develop explanations working for themselves. These explanations can be of any type, the most important is that they shall be aimed to reach the maximum possible interpretability of the AI system by its developers. Yet, some AI models are inherently opaque and cannot be fully explained. We argue that all the possible measures to explain these models shall be taken, but some degree of opacity might be accepted if the benefits of the use of AI application overweigh this opacity risk. In this case, even more attention shall be paid to other transparency and accountability measures.

In addition to the inherent opacity of some AI models, AI providers might have other limitations for explanations to be developed. The availability and the extent of explanations depend on a diverse set of factors that need to be evaluated at the insider layer of transparency and then eventually decided at the internal layer in cooperation with AI users. The factors include, for example, the availability for explanations^44^ and the quality^45^ of the data used in the AI's development and training process, different automated explanations techniques available for the model in question,^46^ legislative requirements (already existing^47^ or to be adopted in the future^48^). These limitations shall be considered in the whole risk-benefit analysis of the AI system in question depending on the purposes, quality, and other features of that system. The functions of transparency shall also be kept in mind for evaluation and deciding on its limitations.

Besides enabling the AI providers to perform their transparency duties toward healthcare providers and patients, the main function of insider transparency is to guarantee the safety and quality of AI devices to the maximum possible extent. This function of transparency is very important for any AI-based device and crucial for those that have interpretability limitations.

### Medical Devices Framework as the Foundation for AI's Insider Transparency

The MDF establishes a rather extensive list of obligations that benefit the safety and quality of the devices. That includes, *inter alia*, drawing up “the records and reports demonstrating appropriate conformity assessment activities” (MDR, Annex VII, art 3.2.4), building the system of recording incidents with a device and its adverse effects (MDR, art. 80), as well as providing quality records as a part of the quality management system (MDR, Annex IX, Chapter I, art 2) to record clinical investigations [MDR, art 72(3)] and data about safety and quality of the device during its whole life cycle, including post-market surveillance [MDR, art 83(2)]. In addition to that, the mentioned recording obligations shall enable notified bodies to carry out the relevant audits and reporting about them (MDR, Annex VI, Part A, 4.6). Documentation of the whole process of the device's development, its risk management system, conformity assessment, and post-market surveillance is also required under the current framework. This list of measures corresponds to the transparency activities identified through the analysis of law (records keeping, documentation, and auditing)^49^ and shall be considered part of the transparency system at the insider level. Information provision established at the internal transparency level also works for helping AI providers hold accountability. These insider transparency measures are presented in Table 3.

**Table 3:** Summary of the MDF requirements (illustrated by the MDR) relevant to AI' insider transparency measures.

Who to whom:	AI providers to themselves;
When:	During the whole life cycle of AI-device;
What:	– Information provision (as specified at the internal transparency level); – Development of explanations for AI systems; – Documentation of the whole process of the device's development, its risk management system, conformity assessment and post-market surveillance; – Keeping the records of: • Carrying out of conformity assessment procedures; • Incidents and adverse effects of a device; • Quality management and reports; • Clinical investigations; • Data about devices' safety and quality.
How:	Records-keeping and documentation shall be carried out in the way that enables notified bodies to audit the activities of AI provider and verify the quality and safety of AI devices. Explanations shall be provided to the maximum technically possible extent and in way that enables further tailor explanations to users. Information shall be provided as specified at the internal transparency level.
Why:	• To hold AI providers accountable; • To ensure safety and quality of AI-devices.

In summary, the MDF establishes a rather extensive list of transparency measures required from AI providers, both at the internal transparency level (toward users of AI systems) and at the insider level (toward AI providers themselves). These measures correspond to the ones identified before from the analysis of law and thus enable to build the transparency system that is discussed in Section Summary and Discussions.

## Summary and Discussions

### Transparency of AI in Healthcare: Context-Specific and Role-Specific Multilayered System of Accountabilities

In this article, we suggest that AI's transparency in healthcare shall be viewed as the multilayered system of measures. Nowadays, discussions about AI's transparency focus on the relevant obligations of AI providers, mostly on the requirement to ensure AI's interpretability. This task is challenging not only due to technical limitations of black-box models but also because interpretability is not the fixed parameter and in the end is evaluated by its addressee. That is why interpretability and more generally transparency shall be context specific and role specific. This approach enables to shape transparency-related obligations based on the roles of the involved subjects and their expectations from transparency in the specific domain. These roles and expectations are also influenced by the applicable legal frameworks and taking them into consideration allows to better implement the AI's transparency measures into the accountability system already existing in the domain.

### Layers of AI's Transparency in Healthcare: External, Internal, and Insider

For the use of AI applications in healthcare, we suggest considering three layers of transparency: external (from physicians toward patients), internal (from AI providers toward physicians), and insider (from AI providers toward themselves). All the layers are interconnected and influence each other. For example, the transparency needs of patients shape the transparency expectations of healthcare professionals from AI providers. At the same time, AI providers cannot explain more than they understand themselves, and so limitations on the insider level dictate the scopes of transparency at the internal and external layers. These limitations arise out of the black-box issue of AI – the lack of possibilities to understand the inner working of algorithms and to fully explain how the specific input turned to the specific output.

### Considerations for the Black-Box Issue of AI

To deal with the black-box issue of AI, we propose several considerations. First, we argue that AI technologies that have this issue shall not be banned from usage even in high-risk areas such as healthcare. The European Commission, despite its vague determination of transparency requirement^50^, seems to support this approach because it includes in the list of allowed AI technologies all the types that are usually described as black boxes (EC Proposal for the AI Act, Annex A). It means that the regulator does not limit the usage to only inherently interpretable algorithmic models, although some data science scholars promote the opposite approach (Rudin, 2018).

Second, despite the technical challenge, AI providers have to take the best possible explainability measures, both for themselves and for other involved subjects. This area of research shall be promoted to motivate data scientists to progress in it and improve the technical facilities to explain AI. For that, the requirement to use state-of-the-art explainability techniques shall be part of the conformity assessment process.

Third, the level of algorithmic opacity that cannot be technically solved at the moment might be considered an acceptable risk subject to its careful evaluation and demonstration that the benefits of AI's use in the specific healthcare application overweigh it. This approach corresponds to the one already existing in the healthcare domain and, more specifically, to the framework that regulates the market authorization of medical devices. The world of no risk is impossible, and it is especially true for the diagnosis and treatment of individuals who are unique and can differently respond to medical actions. We cannot always accurately predict the outcomes of medical treatment, and even more, sometimes we cannot explain the health conditions of the specific person (diagnose him) or explain why his treatment did not help. In this sense, some level of opacity always exists in healthcare, and expecting it from AI devices otherwise is unrealistic. In this case, the requirement of state-of-the-art explanations also works in another direction – it enables AI providers to justify the acceptance of some level of opacity (because the technologies that solve it do not exist).

Finally, the suggested vision of transparency as a multilayered system enables to balance the technical opacity of algorithms by strengthening other transparency measures from AI providers and other involved subjects. This approach corresponds to the vision existing in law and thus makes it more feasible to implement a transparency system into the relevant accountability frameworks.

### Transparency as an Umbrella Category and a Legal Principle

Based on the analysis of law, we have identified that transparency as a broad concept can include different measures such as explainability, interpretability, information provision, traceability, auditability, records keeping, and documentation. The measures that are directly related to solving the “black-box” issue of AI are interpretability and explainability. Through the analysis of literature in the technical domain, we suggest that the concepts shall correlate as follows: transparency is an umbrella category and covers the whole process of AI's use and development; explanations as the actions of involved subjects (of both technical and non-technical natures) are one of the tools to reach interpretability – the state when explanations help addressees to reach their legally protected goals. Considering the technical limitations to provide the full set of explanations, other transparency measures listed above (such as information provision, traceability, auditability, records keeping, and documentation) are to be used as complementary tools to reach the interpretability of the involved subjects and the transparency of the whole process.

In addition to the implementation of these tools and measures, data governance and data management, more generally, shall also be linked more firmly to transparency. The European legislator^51^ includes the identified measures in the AI framework, and in the previous policy documents, most of them were associated with transparency and accountability (including documentation and records keeping). However, the EC Proposal for the AI Act directly associates transparency only with interpretability and communication.

We argue that the further version of the legislative proposal for AI Act shall establish a clear hierarchy between the transparency-related concepts. We submit that the transparency of AI shall be established as an umbrella concept and a legal principle of AI's development and use. The other neighboring concepts specified above shall be enacted as the measures supporting the realization of the principle.

### Currently Applicable Legal Frameworks and Their Ability to Ensure AI's Transparency in Healthcare

We analyzed the legal frameworks that are applicable for the development and use of AI in healthcare to see what kind of transparency requirements are established there. The summary is presented in Annex II, and it is informative guide for AI providers on what is expected from them during the AI lifecycle and what are the roles of other subjects. The table also demonstrates that the applicable legislation is rather detailed in establishing the transparency measures. Legal scholars submit that if interpreted properly, “Medical Devices Framework might provide extremely detailed, extensive and strong obligations of explanation for manufacturers of medical devices relying on AI” (Kiseleva, 2020b; Lognoul, 2020). The amount of AI applications that are already authorized under the current legal framework (even before the AI Act is adopted) proves this argument. We support this vision and, in this article, suggested how the transparency-related obligations in the MDF can be specified for AI-based medical applications to properly ensure transparency in this context.

### Suggestions to Improve the Current Legal Frameworks to Facilitate AI's Transparency in Healthcare

However, the vision of AI's transparency as a multilayered system enabled us to discover the gaps in the current frameworks and to develop suggestions to fill them in. These suggestions mostly concern the role of AI's user – healthcare professional.

First, we argue that AI's external transparency shall be always tailored to the needs of the specific patients and this tailoring is always conducted by a healthcare professional. Physicians have to decide how to comply with the medical consent requirement in a way personalized to a patient based on the information and tools they have. It means that although we shall establish the minimum external transparency obligations with regards to AI, physicians define how to make it completed. In the end, explanations are generated by humans to humans.

Second, because of the increased role of healthcare providers with regards to AI's transparency, they have to be provided with more transparency measures. Similar to external transparency, at the internal layer, transparency shall be tailored to the needs and expectations of physicians (which are influenced by their obligations relevant to medical consent and by the need to hold accountability for their treatment and diagnosis decisions). At this level of transparency, AI providers are its addressors and thus are accountable for the relevant obligations. However, data scientists are not necessarily the experts in the domain and thus might have difficulties in deciding what serves as a good explanation for healthcare professionals. Based on the interpretability evaluation metrics suggested by F. Doshi-Velez and B. Kim and, more specifically, on the application-grounded evaluation, we argue that the quality of explanations developed by AI providers to be supplied together with the device in question shall be assessed by healthcare professionals specializing in the area of the device's use. To organize this type of involvement and to make it independent from AI providers, some sort of controlling bodies representing healthcare professionals for their participation in the AI-device evaluation can be created. The establishment of this type of bodies can be introduced in the future AI Act.

## Conclusion

This paper is the interdisciplinary research to define, organize, and discuss efforts around AI's transparency in the healthcare domain. In Section AI's Transparency: Between Law and Computer Science, we first identified what kind of actions are associated with the notion of transparency in the EU legislation and AI policy documents. We further conducted the review of the literature in computer science to see how the concepts of AI's transparency, explainability, and interpretability can correspond to each other. This analysis in two fields resulted in establishing a hierarchy between transparency and neighboring concepts such as explainability, interpretability, information provision, traceability, auditability, records keeping, documentation, data governance and management. We submit that transparency shall be considered the highest concept in this hierarchy with other concepts as the measures to achieve it. In Section Functions and Types of AI's Transparency in Healthcare, we placed the analysis of AI's transparency in the healthcare context. The contextual investigation of AI's transparency allowed us to identify its functions and types in healthcare. It also enabled us to suggest the AI's transparency as the system of accountabilities for involved subjects that shall take into consideration the risk-benefit approach already existing in healthcare. In Section External Transparency of AI in Healthcare: Toward Patients, we carried out the analysis of the medical informed consent requirement – the legal framework applicable to the external layer of transparency. In Sections Internal Transparency of AI In Healthcare: Towards Healthcare Providers and Insider Transparency Of AI In Healthcare: Towards AI Developers, we explored the MDF that is the main legislative source that regulates the internal and insider levels of AI's transparency. In Section Summary and Discussions, we submitted the main findings of the paper and summarized its results.

The analysis of legal frameworks that regulate several layers of AI's development and use in healthcare, at different stages and in different relations, contributes to the state of the art. Most of the legal scholars focus their attention on either the challenges of medical informed consent in AI context or on possible issues for AI applications under the MDF. In this article, we cover both frameworks and establish links between them with regards to transparency requirements they set. This analysis is reinforced by another contribution of the paper – interdisciplinary research on AI's transparency and related concepts. Together, it resulted in the vision of transparency as the multilayered system of accountabilities for the involved subjects. It also enabled us to discover the existing gaps in the system and develop suggestions to fill them in. By taking into consideration the risk-management system that exists in healthcare, we suggest that some level of algorithmic opacity can be deemed as an acceptable risk subject to its balancing with other transparency measures and the benefits of AI's use.

## Author Contributions

AK holds the first and senior authorship, wrote the first draft of the manuscript, and prepared the final version of the manuscript. PD revised and updated the manuscript and contributed to its legal expertise. DK revised and updated the manuscript and contributed to its technical expertise. All authors contributed to manuscript revision, read, and approved the submitted version.

## Funding

This research was funded and supported by the EUTOPIA Ph.D. co-tutelle program 2020. This program is co-funded by the Erasmus+ Program of the European Union and promotes scientific excellence, research collaboration and academic mobility. Grant number and name: OZRIFTM4 (Balancing transparency of AI in healthcare with safety and quality. A legal and technical perspective).

## Conflict of Interest

The authors declare that the research was conducted in the absence of any commercial or financial relationships that could be construed as a potential conflict of interest.

## Publisher's Note

All claims expressed in this article are solely those of the authors and do not necessarily represent those of their affiliated organizations, or those of the publisher, the editors and the reviewers. Any product that may be evaluated in this article, or claim that may be made by its manufacturer, is not guaranteed or endorsed by the publisher.

## Footnotes

^1^This includes, for example, IBM Watson Health (https://www.ibm.com/watsonhealth) and Google Deep Mind [that was merged with Google Health (https://deepmind.com/blog/announcements/deepmind-health-joins-google-health)]. More on the use of AI in healthcare, see in Astromské et al., 2021.

^2^The controlling body that authorizes medical devices to be placed on the market is the FDA (US Food and Drug Administration, see here: https://www.fda.gov/about-fda/fda-basics/what-does-fda-regulate) and in the EU-notified bodies established under the national law of an EU Member State [MDR (Regulation, 2017a, Annex VII, art. 1)].

^3^The scope of the EU Medical Devices Framework (MDF) covers software used for medical purposes defined in the framework. Thus, most of the AI applications used for medical purposes are covered by the MDF (more details are in Kiseleva, 2020a, and in Kiseleva, 2021b).

^4^AI models vary in the level of their inherent lack of transparency (starting from the opaquest ‘black-box’ models to less opaque ‘gray-box’ and ‘white-box’ models). However, all of them could have some limitations in the level of transparency they offer, based on different factors (such as amount, type, balance, and representativeness of data used to develop and train AI – this might limit the comprehensibility of the process/model or the results even for ‘white-box’ models). Although in this paper we discuss the possible solutions mainly around the issue of ‘black-box’ models in AI, the general approach toward AI's transparency in healthcare developed in this paper is applicable and relevant to any type of AI model.

^5^The number of authorized AI-based medical applications as identified by Urs J Muehlematter et al., 2021 is the additional proof of applicability of the currently existing MDF to AI-based medical applications.

^6^The legal perspective of this paper includes an analysis of the EU law. However, the common interdisciplinary approach to AI's transparency in healthcare can be applied (subject to the relevant specifications) to legal systems in other countries such as the USA. For example, the MDF and the relevant procedures in the USA are similar to the EU ones. Formally, the framework also covers software and thus can be applied to AI-based devices (Kiseleva, 2020a). Rules of informed consent are also similar in their core. Thus, the technical perspective of the paper, the transparency taxonomy and architecture, and overall general methodology for legal analysis can be applied to the non-EU context.

^7^Although we refer further to the research to technically solve the issue of AI's opacity, this scope of the analysis is limited due to the interdisciplinary nature of the paper.

^8^We use the term ‘access to information’ rather than ‘information provision’ because the latter means active behavior to provide information while the right to information can include the obligation to refrain from prevention to receive the information or the obligation to provide it actively or passively.

^9^All the transparency actions can be relevant to all three types of objects: data, knowledge, and information.

^10^Holzinger and Mueller suggest mapping explainability (in a technical sense) to causability – ‘the measurable extent to which an explanation of a statement to a human expert achieves a specified level of causal understanding with effectiveness, efficiency, and satisfaction in a specified context of use' (Holzinger and Mueller, 2021).

^11^The dependency of explanations and more generally of transparency on the context and roles of the involved subject will be further elaborated in Sections Functions and Types of AI's Transparency in Healthcare, External Transparency of AI in Healthcare: Toward Patients, and Internal Transparency of AI in Healthcare: Toward Healthcare Providers of this paper.

^12^‘In essence, data governance concerns the deployment of the right mixture of process, technology, and personnel to govern the input, storage and usage of data to achieve the objectives of the system where data is used' (Sundararajah). It includes data stewardship (promoting accountability by assigning stewards/custodians to relevant datasets), data accessibility (facilitating the availability of data for relevant stakeholders), data security, quality control, and knowledge (preserving and improving data knowledge by ensuring documentation of data systems and related processes are kept up to date) (Sundararajah, 2021).

^13^‘Making the opaque and hidden visible, creates truth and truth enables control, which serves as a ‘disinfectant’ (Rieder and Hofmann, 2020, reference to Brandeis, 1913) capable of eliminating malicious conduct’ (Rieder and Hofmann, 2020).

^14^It concerns all the procedures in the medical area starting from clinical trials and authorization of medicines and medical devices finishing with monitoring (meaning collecting and analyzing new data) of patients during and after their treatment. For example, data about verification, risks, and benefits of the specific medicine shall be shared with physicians so that they can prescribe it to patients in a safe and efficient manner. In turn, physicians shall properly inform patients about the prescribed medicine so that patients can tell the physicians if they have any contraindications which, in turn, enables preventing risks and side effects for them. Overall, access to and understanding of all the relevant information and knowledge by all the concerned subjects makes their decision-making safer and more efficient.

^15^Carvalho et al. used this phrase in relation to interpretability, but we extend this reference to transparency in general (Carvalho et al., 2019).

^16^The ability to make an informed decision is closely related to the other goal that transparency helps to achieve for patients – the realization of individual rights. The right to consent or refuse medical treatment patients are properly informed about is itself the right that shall be protected. However, the EU Charter of Fundamental Rights links the right to informed medical consent with the broader rights – the right to respect for physical and mental integrity (Charter of Fundamental Rights of the European Union, 2012, art 3). In the broadest sense, this right is, in turn, relates to human dignity with is protected under article 1 of the Charter. This link to the very fundamental right highlights the importance of enabling the choice for patients which, in turn, is achieved through transparency. Legal scholars also highlight the importance of the fundamental rights framework for the use of AI in medicine and healthcare (Schneeberger et al., 2020; Stoger et al., 2021).

^17^External and internal healthcare transparencies are inextricably connected. Stressing it out, external transparency cannot exist without the internal one. It means that the focus of healthcare policy makers and stakeholders shall start from ensuring transparency to patients but ultimately leads to creating the system where transparency is guaranteed to healthcare providers. While internal transparency is valuable itself (it enables to make the choices of healthcare providers more informed and thus to improve them), one of the main functions of internal transparency shall be ensuring the external one.

^18^See above Section Functions of AI' Transparency in Healthcare.

^19^Due to issues with AI's transparency, some legal scholars suggest that for the algorithmic governance in privacy and data protection, priority shall be given to accountability instead of transparency (De Hert and Lazcoz, 2022). While generally agreeing with the importance of accountability, in the context of this paper, we see accountability as an architecture to build a transparency system rather than the prevailing concept.

^20^Multilayered approach toward transparency (or explainability) has received its recognition in legal scholarship. With regards to explanations in the GDPR, Kaminski and Malgieri suggested the layers of individual, group, and systemic explanations (Kaminski and Malgieri, 2020). Similarly, Zarsky distinguished groups of data recipients in a transparency system: general public, internal, and external institutions, and affected individuals (Zarsky, 2013). In this paper, the layers of transparency are placed in the healthcare context and defined by more specific roles of the relevant actors. In addition to that, the layers themselves guide the choice of applicable legal framework at that level – which extends the instrumentality of the suggested multilayered approach.

^21^The accountability system based on posing the said questions is described by Bovens, Schillemans, and Goodin (Bovens et al., 2014). A similar question-based approach was suggested by Kaminski with regards to explanations in the GDPR (Kaminski, 2019) and she further applied it for transparency in algorithmic accountability (Kaminski, 2020).

^22^For example, transparency measures that are needed for patients to make their informed choice about their health and body will differ from the ones that are needed for AI providers to ensure the general safety and quality of AI system. Healthcare providers need to make their choice about treatment options for a patient and at the same time to ensure the safety and quality of the AI's outcome concerning the patient in question.

^23^Transparency rules for AI shall be developed taking into consideration not only existing transparency-related requirements (such as the rule of informed medical consent) but also all the legal obligations imposed to the involved stakeholders and the relationships between them. Because one of the functions of AI's transparency in the healthcare context is ensuring the safety and quality, the existing rules on that matter shall be explored to understand what is expected from AI providers under the current framework and how it works for AI's transparency.

^24^In this paper, we suggest the general approach to look at AI's transparency in healthcare (not only explainability in a technical sense) on correspondence to all its benefits, not limited to accuracy. This approach enables to consider the explainability-accuracy trade-off existing in the AI context but allows to look at the problem wider based on the extended interdisciplinary vision on transparency. For the detailed analysis of the trade-off between explainability and accuracy from a legal perspective (with technical case study and literature), including contract and tort law, see Hacker et al., 2020. In the medical context, they suggest three factors that shall be considered for the use of ML with an acceptable level of accuracy: ‘if ML models are shown to be consistently superior to human decision making, if they can be reasonably integrated into the medical workflow, and if they are cost-justified’ (Hacker et al., 2020).

^25^In this scenario, viewing transparency as the system that is not limited to only interpretability (where the risk of not achieving it exists) enables to minimize the overall number of risks through other, more feasible measures of transparency such as explanations, data governance, audit, records keeping, and documentation.

^26^The group of recipients of medical care is broader than patients (who can be understood as only those who are admitted to hospitals), but for the purposes of this paper, we use the word ‘patients’ to refer to everyone who receives medical care from healthcare professionals in healthcare organizations.

^27^Considering the scope of the paper, we limit the analysis with the guidance on answering the posed questions under the legal and technical perspectives of transparency-related concepts explored in Section AI's Transparency: Between Law and Computer Science.

^28^These measures correlate to the transparency-related concepts identified in Section AI's Transparency: Between Law and Computer Science.

^29^Which is required under the rule of the medical informed consent (see the Table 1).

^30^This submission is made because the current legislative requirement of informed medical consent does not explicitly include the explanation element.

^31^Similarly, Zarsky suggested transparency stages for AI's development and use: data collection and aggregation; data analysis and examining model uses (*ex-ante* and *ex-post*) (Zarsky, 2013).

^32^Explanations by design are also differentiated from the explanations of AI developers provided in non-technical form (such as clarifications to AI users).

^33^For example, J. Petch identified the following explainability techniques in cardiology: variable importance methods (feature importance methods that generate explanations by calculating the statistical contribution of each variable to a model performance), surrogate methods (explain a black-box model by developing a new interpretable model, such as a logistic regression or a short decision tree, on the predictions of the black-box model), and visualization methods (illustrate relationships between an outcome and a set of variables of interest. However, the authors accept the limitations of these techniques (Petch et al., 2021). Guidotti et al., in their detailed survey, classified methods for explaining the black-box models based on several factors: the type of problem faced, the type of explanator adopted to open the black box, the type of black-box model that the explanator is able to open, and the type of data used as input by the black-box model (Guidotti et al., 2018).

^34^As required under the rule of the medical informed consent (see the Table 1).

^35^Under the MDF, performance is the ability of a device to achieve its intended purpose as stated by the manufacturer [MDF, art. 2(22) and IVDR, art. 2(39)]. The two acts included in the MDF are different in their specification of accuracy with relation to devices' performance: the IVDR explicitly includes accuracy in the elements of the device's performance [see the detailed analysis in Kiseleva and Quinn (2022)] and the MDR requires the provision of sufficient accuracy for the intended purpose and indication of accuracy limits only for diagnostic and measurement devices [MDR, Annex I. 15(1)].

^36^Similarly, Friedrich et al. indicated that ‘although medicine is one of the oldest sciences, mechanisms through which interventions work are sometimes not known or not well understood. For example, clinicians utilized aspirin for centuries without understanding exactly how it worked (and the discovery of its mechanism of action was considered worthy of a Nobel Prize in 1982)' (Friedrich et al., 2022).

^37^The tailoring shall also depend not only on the patient and her individualized treatment process but also on the AI application in question and its level of opacity and self-learning.

^38^While we explored this framework in the previous section, we do not focus on it here, but keep in mind its influence on the internal transparency, too.

^39^As mentioned in the introduction, the MDF consists of two legislative acts: the MDR (Regulation, 2017a) and the IVDR (Regulation, 2017b). These two acts are rather similar in their procedures and concerned subjects (in relevance to the scope of this paper and in correlation to transparency). For the simplicity of further analysis, the MDR will be used as a point of reference.

^40^Despite the lack of explicit transparency requirement in the MDF, this conclusion is possible due to consideration of internal transparency as one of the tools to ensure the AI's safety and quality – the scope of the framework.

^41^See the clarification of this term in Section Measures of External Healthcare Transparency in AI Context of this paper.

^42^In this paper, we do not propose the specific measure to achieve the application-grounded evaluations of explanations and interpretability because it shall be always contextualized (not only for healthcare context but also for the specific AI application). However, a useful example of such a measure is the system causability scale (SCS) suggested by Holzinger et al. (2020).

^43^See the experiments on human-in-the-loop evaluations of explanations of different styles here: (Shulner-Tal et al., 2022).

^44^Data that have been used in the AI model training/prediction process might not be sufficient for providing explanations and/or its use for explanations might be limited (due to privacy and data protection issues, for example).

^45^For example, the quality of data might be sufficient for the use in the prediction process, but not enough for explanations (Holzinger et al., 2017).

^46^Some ‘black-box' models might require the use of ‘white-box’ models to generate explanations (Kenny et al., 2021).

^47^Already existing legislative requirements mainly include the ones described in this paper (the requirement of the informed medical consent, MDF), but can also include the need to comply with the GDPR (these requirements are not the focus of this paper but are extensively explored by legal and other scholars (see, for example, Malgieri, 2021; Mourbya et al., 2021; Hamon et al., 2022).

^48^For example, if the future version of the AI Act establishes the strict means of explanations (such as, always requiring automated explanations), that might pose additional technical limitations. Automated explanations available for the specific model in question might be either too generic (and thus of limited usability) or too many (and thus blur the picture) or too few (and thus inadequate).

^49^See Section Views on AI's Transparency: Law and Policy.

^50^More on that in Kiseleva (2021) and Kiseleva (2021a).

^51^As we saw in Section Transparency in the EU Policy Documents on AI.
