# Supplementary material for: Transparency of AI in Healthcare as a Multilayered System of Accountabilities: Between Legal Requirements and Technical Limitations
Source: Front Artif Intell. 2022 May 30;5:879603. doi: 10.3389/frai.2022.879603 (PMC9189302; doi:10.3389/frai.2022.879603)
Supplement: Supplementary file 1 [file Data_Sheet_1.zip › Annex I.pdf]

ANNEX I
List of EU laws articulating the concept of transparency (non-exhaustive)

Commission Directive 2006/111/EC of 16 November 2006 on the transparency of financial relations between Member States and public undertakings as well as on financial transparency within certain undertakings, OJ L 318/17, 17/11/2006

Directive 2004/109/EC on the harmonisation of transparency requirements in relation to information about issuers whose securities are admitted to trading on a regulated market as of December 15, 2004, *OJL 390*, 39.12.2004, p. 38-57

Regulation 2015/2365 on transparency of securities financing transactions as of November 25, 2015, OJL 337, 23.12.2015, p. 1-34

Regulation 1227/2011 on wholesale energy market integrity and transparency as of October 25, 2011, OJL 326, 8.12.2011, p. 1-16

Regulation 2017/352 establishing a framework for the provision of port services and common rules on the financial transparency of ports as of February 15, 2017, OJ L 57, 3.3.2017, p. 1-18

Council Directive 89/105/EEC relating to the transparency of measures regulating the pricing of medicinal products for human use and their inclusion in the scope of national health insurance systems as of December 21, 1998, OJ L 40, 11.2.1989, p. 8-11

Directive 2019/1152 on transparent and predictable working conditions in the EU as of June 20, 2019, OJ L 186, 11.7.2019, p. 105-121

Regulation (EU) 2019/1381 of the European Parliament and of the Council of 20 June 2019 on the transparency and sustainability of the EU risk assessment in the food chain and amending Regulations (EC) No 178/2002, (EC) No 1829/2003, (EC) No 1831/2003, (EC) No 2065/2003, (EC) No 1935/2004, (EC) No 1331/2008, (EC) No 1107/2009, (EU) 2015/2283 and Directive 2001/18/EC, OJ L 231, 6.9.2019, p. 1–28

Regulation (EU) 2019/1150 of the European Parliament and of the Council of 20 June 2019 on promoting fairness and transparency for business users of online intermediation services, OJ L 186, 11.7.2019, p. 57-79

Directive (EU) 2019/1024 of the European Parliament and of the Council on open data and the re-use of public sector information as of 20 June 2019, OJ L 172, 26.6.2019, p. 56-83
