# Supplementary material for: Transparency of AI in Healthcare as a Multilayered System of Accountabilities: Between Legal Requirements and Technical Limitations
Source: Front Artif Intell. 2022 May 30;5:879603. doi: 10.3389/frai.2022.879603 (PMC9189302; doi:10.3389/frai.2022.879603)
Supplement: Supplementary file 1 [file Data_Sheet_1.zip › Annex II.pdf]

ANNEX II. SUMMARY OF THE EXISTING LEGISLATIVE TRANSPARENCY-RELATED REQUIREMENTS FOR AI-BASED MEDICAL DEVICES

	**EXTERNAL**	**INTERNAL**	**INSIDER**
LEGAL FRAMEWORKS	requirement of the informed medical consent	requirement of the informed medical consent and the Medical Devices Framework	Medical Devices Framework
**WHO TO WHOM**	Healthcare providers to patients	AI provider to healthcare professional (AI-based device's user) and patient	AI providers to themselves
**WHEN**	before health intervention	when an AI-based device is placed on the market and being used	during the whole life cycle of AI-device
**WHAT**	information as to the purpose and nature of the intervention as well as on its consequences and risks	Providing of information about:<br>•instructions for the appropriate use of a device;<br>•safety and quality of a device;<br>•expected clinical benefits;<br>•device's performance characteristics;<br>•information allowing assessment of the device's suitability;<br>•residual risks, contra-indications, and any undesirable side-effects;<br>•information allowing being informed about risks, contra-indication, and undesirable side-effects;<br>•requirements for special facilities, or special training, or particular qualifications of the device user and/or other persons;<br>•specifications the user requires to use the device appropriately	Information provision (as specified at the internal transparency level);<br>Documentation of the whole process of the device's development, its risk management system, conformity assessment and post-market surveillance;<br>Keeping the records of:<br>•carried out conformity assessment procedures;<br>•incidents and adverse effects of a device;<br>•quality management and reports;<br>•clinical investigations;<br>•data about devices' safety and quality.
**HOW**	appropriate information	information relevant to the user and tailored to his technical knowledge, experience, education, or training. Instructions for use shall be written in terms readily understood by the intended user and, where appropriate, supplemented with drawings and diagrams	Records-keeping and documentation shall be carried out in the way that enables notified bodies to audit the activities of AI provider and verify the quality and safety of AI devices.<br><br>Information shall be provided as specified at the internal transparency level.
**WHY**	to enable free and informed consent/rejection of the intervention (make the choice about it)	•to enable healthcare providers making choices, including diagnosis and treatment ones;<br><br>•to hold healthcare providers accountable, including with regards their transparency obligations towards patients;<br><br>•to ensure safety and quality of AI-devices, both at the general level and with regards to the specific patient.	•to hold AI providers accountable;<br><br>•to ensure safety and quality of AI-devices.
